# Characterizing Meat- and Milk/Dairy-like Vegetarian Foods and Their Counterparts Based on Nutrient Profiling and Food Labels

**DOI:** 10.3390/foods12061151

**Published:** 2023-03-09

**Authors:** Noelia María Rodríguez-Martín, Patricia Córdoba, Beatriz Sarriá, Vito Verardo, Justo Pedroche, Ángela Alcalá-Santiago, Belén García-Villanova, Esther Molina-Montes

**Affiliations:** 1 Group of Plant Protein, Department of Food and Health, Instituto de la Grasa-CSIC, Campus Universitario Pablo de Olavide, Edificio 46, Carretera de Utrera Km. 1, 41013 Seville, Spain; 2 Department of Nutrition and Food Science, Faculty of Pharmacy, University of Granada, 18071 Granada, Spain; 3 Department of Metabolism and Nutrition, Institute of Food Science, Technology and Nutrition (ICTAN-CSIC), Spanish National Research Council (CSIC), José Antonio Nováis 10, 28040 Madrid, Spain; 4 Department of Nutrition and Food Science, Faculty of Pharmacy, University Complutense of Madrid, 28040 Madrid, Spain; 5 Institute of Nutrition and Food Technology (INYTA) ‘José Mataix’, Biomedical Research Centre, University of Granada, Avenida del Conocimiento s/n, 18071 Granada, Spain; 6 Instituto de Investigación Biosanitaria ibs.GRANADA, 18012 Granada, Spain; 7 CIBER de Epidemiología y Salud Pública (CIBERESP), 28029 Madrid, Spain

**Keywords:** vegetarian, vegan, plant-based food, animal-based food, Nutri-Score, nutritional quality

## Abstract

Vegetarian foods are plant-based (PB) foods, often perceived as healthier foods than animal-based (AB) foods. The objective of this study was to analyze the nutritional quality of a set of PB foods (meat, milk and dairy products) marketed in Spain, and to compare their nutrient profiles with respect to some AB counterparts. Nutritional information per 100 g or mL, ingredients, and nutritional declarations, as well as the Nutri-Score, NOVA, and Eco-Score of each food were collected from Open Food Facts. Differences in the nutrient compositions between PB foods and their counterparts, and between the different groups of PB foods, were assessed at a 5% significance level. A total of 544 PB foods and 373 AB foods were identified. Overall, PB foods had a higher median content of fiber and carbohydrates, but a lower amount of proteins (except PB “meat” analogues: 14 g) and saturated fats (except PB “cheese alternatives”: 12.5 g), than the AB counterparts (*p* < 0.05). PB “milk alternatives”, particularly oat “milk”, showed a higher median content of total carbohydrates (8 g) and sugars (5.5 g) compared to cow milks (4.7 g carbohydrates/sugars, on average; *p* < 0.001). PB “meat alternatives” also had a significantly higher value of carbohydrates (9 g) than AB meats (2 g, on average; *p* < 0.001). PB foods were mostly classified as Nutri-Score A and B (86%). However, more than half of them were of NOVA groups 3 and 4. Thus, there is a great diversity of PB meat and milk/dairy product alternatives on the Spanish market. Despite being products of good nutritional quality compared to AB foods, they also carry drawbacks that could have an impact on nutritional health.

## 1. Introduction

Plant-based (PB) foods have undergone a creeping trend in consumption over the last few years. Indeed, the increasing demand for PB foods, fueled by the need to find new alternatives for health-conscious consumers, has led the food industry to spread the production of the so-called vegetarian or veggie foods [1]. Consumers have nowadays far more choices of vegetarian-like foods than ever before. For instance, milk-free dairy products, or meatless burgers, among others, are readily available in the market. Several facts illustrate this phenomenon: the European project SMART PROTEIN reported that PB food sales increased by 49% from 2018 to 2020 in Europe [2]. In Spain, those sales entailed 20% of the value in the market. This report also showed that the PB sector in Spain was dominated by vegetable drinks, with oat beverages being the most important ones, followed by PB “meat alternatives” and vegetable yoghurt, of which sales increased by 55%. In addition, during a survey carried out between 2021 and 2022 among Spanish people, approximately seven percent of people aged between 20 and 29 reported to follow a PB diet [3]. 

There are three well-established reasons why people may decide to adhere to a vegetarian-like or PB diet: health, animal welfare, and environmental sustainability [4]. Indeed, PB diets have been considered as more sustainable than an omnivorous diet, wherein both plant and animal food sources are eaten [5,6]. Conversely, meat, and animal foods in general, account for nearly 60% of all greenhouse gases from food production [4,7]. 

The fact that a PB diet is considered a healthier diet alternative is supported by some nutritional studies that have shown that vegetarian dietary patterns have an adequate nutrient supply, even during pregnancy, lactation, or in sport nutrition [8,9], except regarding nutrients provided exclusively or overwhelmingly by animal-based (AB) foods [10,11,12]. The healthiness of PB diets is also supported by many studies that have depicted the favorable impact that this dietary pattern has on different health outcomes including cardiovascular diseases and cancer, amongst others [13,14,15,16,17,18,19]. PB diets also seem to promote a healthier microbiota [20,21,22]. However, as aforementioned, some nutrient deficiencies are also likely, most notably in vegan diets, regarding vitamin B12 and iron, but also for omega-3 fatty acids, calcium, vitamin D, and zinc. These potential deficiencies, however, can be alleviated by taking nutritional supplements [10,11,12]. 

The health benefits of plant-source foods in PB diets (legumes, nuts, whole grains, fruits, and vegetables, as well as vegetable oils) can be attributed to the presence of bioactive compounds in these foods [23,24,25]. More precisely, plant bioactives including fiber, sulfur compounds, carotenoids, and polyphenols, present in cruciferous and allium vegetables, tomatoes, green tea, and whole grain cereals, have a well-known antioxidant and anti-inflammatory potential [23,26], while AB foods are poor in these compounds. 

For the above reasons, PB foods can be conceived as healthier than their AB counterparts by the consumer, but this need not necessarily always be the case [27]. Some of these foods can be regarded as ultra-processed since they can be obtained through different combinations of ingredients (soy-protein isolates, vegetable fats, modified starches, malto-dextrins, cosmetic additives such as flavour and colouring enhancers, sweeteners, emulsifiers, and others) and by using several processing methods (for example, extrusion/texturization, hydrolysis, and fermentation) [28,29,30]. In fact, some studies have revealed that vegetarians eat more ultra-processed foods than meat eaters, and that consumption of these foods leads to more detrimental health effects [28]. Regarding the nutritional characteristics of foods of animal and vegetable origin, there are also some key differences to note. For instance, in relation to protein quality, the protein digestibility, the biological value, the net protein utilization, and the amount of essential amino acids are lower in vegetable proteins compared to those of animal origin. Other downsides of vegetable proteins are the presence of anti-nutritional factors that interfere in their absorption, and of potential allergenic proteins. On the other hand, PB foods feature a higher content of carbohydrates, fiber and sugars (in cereal-based foods mostly) than AB foods, whereas the latter add more fats (saturated fats) to the diet than vegetable food sources [23,24]. 

Thus, PB foods can be of different nature, with varied nutrient composition and effects on health [11]. Indeed, PB ultra-processed foods can be high-energy-dense foods, rich in sugars and fats, making their habitual consumption unhealthy. The reverse could also be possible if PB foods involve healthy and natural foods. It is therefore highly relevant to characterize their composition and nutrient content since not all PB foods might be equally healthy from a nutritional point of view. There is also a need to provide information on their nutritional value with respect to AB foods, but there are only a few studies that have compared the nutritional profiles of some PB and AB foods [31,32,33].

We aimed to conduct a comprehensive analysis of the nutritional composition of vegetarian foods available on the Spanish food market, including vegetable beverages and dairy products and PB “meat alternatives”, by collecting the nutritional information, ingredients, and facts reported on their front-of-pack and pack labelling. To complement this analysis, we also considered other information available for the consumer with regard to nutrient profiling models used to classify the nutritional quality, processing degree, and ecological fingerprint: the Nutri-Score, the NOVA index, and the ECO score [34,35,36]. In addition, the nutrient profiles and composition of these foods, as well as the index scores, were compared with that of some of their counterparts (AB meat products, cow milk, and dairy products). 

## 2. Materials and Methods 

### 2.1. Sample Selection

Nutritional data of the food products were obtained from websites of supermarket and food companies, and from Open Food Facts [37]. The latter was the main information source used to retrieve the food products. Open Food Facts is an open data base of front-of-pack nutrition and pack labelling information, which contains complete information on ingredients including additives, allergens, and nutrition facts of food products sold on the market of several countries, including Spain. The website and app offer easy to access information to the consumers, helping them to make better food choices. It is also widely used by scientists and food producers to explore the food system and the nutritional quality of food products [38,39]. However, the information is made up of voluntary contributions of scanned barcodes, pictures, and labels. While this information is checked and regularly updated [37], we used the aforementioned market websites to complement and verify the data.

For the present study, the search was restricted to foods sold on the Spanish market following the inclusion criteria: (1) vegetarian-like foods marketed in Spain, but restricted to meat and milk/dairy product substitutes; (2) food items with complete information in Open Food Facts, or easy to complete with information posted in this and other sources (market websites). The exclusion criteria were: (1) repeated products, e.g., similar foodstuffs but with a different flavour (because their nutritional value is similar); (2) products with incongruent labelling information with respect to the product’s characteristics. 

Additionally, in order to gather some homologous AB foods for the comparative study on nutritional differences between PB and AB foods, we looked for food products of pate and commercial meat products, cheese, yoghurt, and cow milk. For AB yoghurt, cheese, and pate, a restricted sample of items was selected due to product variability on the market. In particular, AB that were similar to the PB ones were selected in an attempt to match food items of both groups. For example, yoghurts with fruits as ingredients were chosen for both sets of PB and of AB foods; i.e., to make PB and AB yoghurts comparable, we selected PB “yoghurt alternatives” with fruits available on the market and an equivalent number of AB yoghurts with fruits. Since the PB “cheese alternatives” were quite heterogeneous, for the AB cheese group we selected items of various types of cheese on the Spanish market (fresh, cured, and semi-cured cheeses), resembling those selected for the PB group. The same was applied for AB pate. To account for high- and low-fat milks, we considered whole milk, as well as semi-skimmed and skimmed cow milk. Similarly, for meat products, we covered red (beef and pork) and white (chicken/poultry) meat. 

A total of 1652 food products, of which 1201 were PB foods classified as veggies, and the remaining potential AB foods to be considered as analogues, were available in Open Food Facts. After the removal of PB foods other than PB “meat”, “milk”, and “dairy product” alternatives, repeated products, products with missing information, or those not complying with the inclusion criteria, a total of 917 food items remained available for the analysis: 544 PB and 373 AB food items.

### 2.2. Data Collection 

The search in the aforementioned online resources was performed between March and May of 2022; additional data on PB beverages was collected in November 2022. Key words used to search in Open Food Facts were: veggie, oat “milk”, soy “milk”, almond “milk”, vegetable “yoghurt”, vegetarian “spreadable”, plant-based “cheese alternatives”, vegetable “paste”, and “tempeh”. For the AB food homologues, the key terms were: cow milk (skimmed, semi-skimmed, and whole milk), pate, cheese, yoghurt, and meat products (chicken, pork and beef hamburgers and sausages). Information was extracted from pictures of the labels provided in Open Food Facts and market websites when needed; thus, the information collected corresponded to that reported by the manufacturer.

The variables collected for every food item were: the commercial name, denomination, portion size, brand of the product, the ingredients (from high to low content), and the nutrition and health claim declarations according to Council Regulation (CE)1924/2006 [40]. For the latter, we collected information on the nutrition claim (high in, source of, etc.) and the amount of the specified nutrient. In addition, we collected information on the presence of indications for gluten and lactose-free products, among others. Secondly, the nutritional information present in the pack labelling was collected according to Council Regulation (UE)1169/2011 [41]: energy (kcal per 100 g/mL and kJ per 100 g/mL), and grams per 100 g or mL of total fat, saturated fat (part of total fat), carbohydrates, sugars (part of carbs, and accounting for natural and added sugars), fiber, proteins, and salt, as well as the list of ingredients. This nutritional information was also collected in terms of Reference Intake values for a healthy adult (daily nutritional requirement of 2000 kcal), according to the aforementioned European labelling rules. In addition, the rating of the product according to the nutrient profiles of Nutri-Score, NOVA, and Eco-Score were collected [34,35,36]. 

All variables were collected through an online form via Google Forms to facilitate data retrieval, sharing, and processing. Three researchers, who were trained to collect all the data under the same standards and protocols, retrieved and reviewed the data (NRM, PC, and EMM).

### 2.3. Data Processing

Several quality controls were applied on the data. For instance, as described before, food items with missing information on nutritional values taken from the front-of-pack nutrition and pack labelling, were completed by re-consulting Open Food Facts and the online market websites. Potentially incorrect values, such as extreme values that may have arisen from data entry or data inconsistencies, were detected by describing the distribution of each variable via box-plots and by sorting values from the lowest to the highest (minimum and maximum values). The values were revised and corrected, where necessary, after verifying the accuracy of the value in all the information sources. In addition, the nutritional data of some food products was checked with published data in Spanish Food Composition Tables to detect potential errors or inconsistencies [42]. Likewise, for the PB foods, the nutritional values of a set of products (N = 100) were checked with those reported in the Spanish Veggiebase [43].

PB food items were grouped in the dataset according to their nutritional characteristics: (a) PB beverages (considered as PB “milk alternatives”), (b) PB “meat alternatives” (“hamburgers” and “sausages”, both sharing similar ingredients), (c) PB spreadable or paste (considered as PB “pate alternatives”), (d) PB “yoghurt”, (e) PB “cheese alternatives”. 

As for the AB products, the following categories were established: (1) cow milk (distinguishing between whole, semi-skimmed and skimmed milk), (2) pate, (3) cheese, (4) yoghurt, (5) and meats (distinguishing by type of meat—beef, pork and chicken, and product—hamburgers and sausages). 

A description of the food products included in each group is provided in Appendix A.

### 2.4. Nutri-Score, NOVA, and Eco-Score Assessment

The values of Nutri-Score of each food item were firstly obtained from the Open Food Facts database, which implements an algorithm to calculate it upon the nutritional data of the front-of-pack labels [44]. Nutri-Score is based on the following classification scheme: A (dark green), B (light green), C (yellow), D (orange), and E (dark orange). To calculate the value, different nutrient profiles are used. The unfavorable are: energy (kJ/100 g), saturated fat (g/100 g), sugars (g/100 g), and salt (g/100 g); whereas the favorable are: the % of fruits, vegetables, legumes, dried fruits and olives, walnut and rapeseed oils, fiber (g/100 g), and protein (g/100 g) [34].

Nutri-Score has been proposed to become mandatory in the near future. Therefore, for food products with missing information on this score (2.5% of all products), we considered the Excel sheet provided by the Spanish Agency of Food Security and Nutrition (AECOSAN) [34]. To apply it, the percentage of fruits, vegetables, legumes, nuts and rapeseed, walnut, and olive oil was taken from the ingredients list. The fiber content was assigned to zero in those food items (*N* = 92, of which 62 were AB food items) without indication for this component on the label after revising the list of ingredients to verify the absence of fiber. Moreover, we double-checked Nutri-Score labelling from Open Food Facts by calculating this score with the AECOSAN tool in the above described set of food products. 

Open Food Facts also served to retrieve the NOVA and Eco-Score values of all items [45]. However, in the case of lack of information for either NOVA or Eco-Score in the pack labeling, these scores were not recalculated by us in order to report the missingness rate of these values. Both NOVA and Eco-Score are not mandatory labels in food products.

The NOVA score is related to the degree of processing of a food product [35]. Foods are classified into four categories according to the nature and industrial process: Group 1 (unprocessed foods or minimally processed), Group 2 (processed by culinary ingredients), Group 3 (processed food), and Group 4 (ultra-processed food, by using cosmetic additives, fractioning of whole foods into substances, and sophisticated technologies). 

The Eco-Score is an indicator that classifies the products into 5 categories (A, B, C, D, E) according to their environmental impact. The factors taken into account are the pollution of air, water, oceans, and soils. In addition, the product life cycle (LCA) was considered [36]. Based on the total score (maximum value = 100), products were classified from A (low impact t) to E (high impact) [46].

### 2.5. Description of Ingredients, Additives, and Claims

As for the other variables, pack labels from Open Food Facts and other websites were used to retrieve information on all ingredients and claims. A complete count was carried out regarding the number of ingredients, the additives, and the nutritional and/or health claims of every food item. For this purpose, keywords (i.e., nutrients) were searched in the dataset. These searches were done in an automated manner since there were specific fields for every variable in the data collection form and dataset. Variables were created to further account in the data analyses for the presence/absence of nutrition claims and of health claims, the presence/absence of gluten or lactose-free indications, the number of ingredients, and the number of additives. The frequency of a certain claim or nutritional indication among all foods, or by food group, was expressed as a percentage rate.

### 2.6. Statistical Analysis

The statistical analysis was carried out using the statistical software R version 4.1.2 [47]; *p*-values less than 0.05 were considered statistically significant. According to the Kolmogorov–Smirnov normality test, all nutritional variables followed a non-normal distribution (all *p*-values < 0.05). Therefore, non-parametric tests were used to perform statistical tests. Continuous variables relative to nutritional information were expressed as median (p50 values) and interquartile ranges (IQR), i.e., p25 and p75 values. For categorical variables (considering food items classified into Nutri-Score, NOVA, and Eco-Score), relative and absolute frequencies were used. Differences in the median content of nutrients of the pack labelling across all PB food groups (for example, PB “milk alternatives” vs. PB “meat alternatives” vs. PB “cheese alternatives”, etc.) or by PB and AB food groups (for example, PB vs. AB food groups: PB “pate alternatives” vs. pate, PB “cheese alternatives” vs. cheese, PB “milk” vs. cow milk of different types, PB “meat alternatives” vs. AB meats of different types) were analyzed by the Kruskal–Wallis test (non-parametric ANOVA test for more than 2 independent sample groups) and the Wilcoxon rank sum test (for two independent sample groups). Post hoc Tuckey test was applied for pair-wise comparisons of PB foods within a PB food group (milks, meat and meat products, dairy products, and others) by their nutrient composition (corrected by the family-wise error rate) to detect significant differences by groups. Chi-Square tests were carried out to evaluate differences among Nutri-Score, NOVA, and Eco-Score from PB or AB food groups. Boxplots and pie charts were used for graphical illustrations of the results using ggplot2 package in R.

## 3. Results

**Figure 1:**
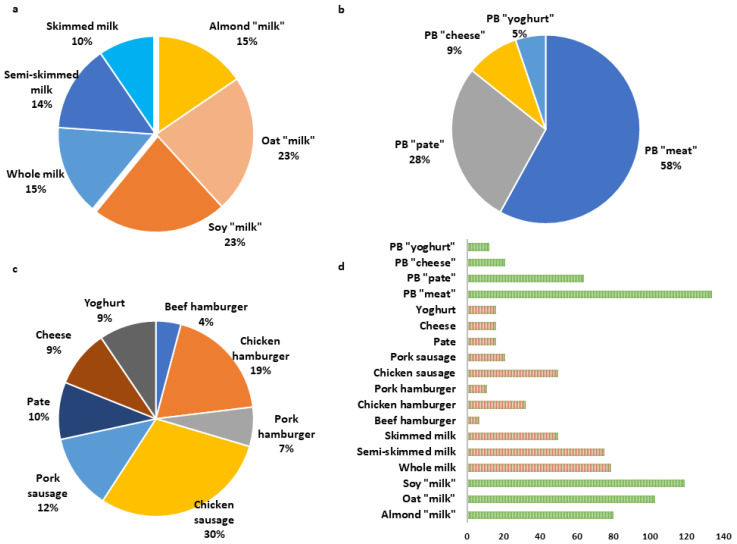
Proportion of plant-based (PB) foods and non-plant-based foods, i.e., AB foods, that were included in this study: (**a**) group of beverages including PB “milk alternatives” (almond, oat, and soy “milk”) and AB milk items (whole cow milk, semi-skimmed, and skimmed milk); (**b**) PB foods without PB “milk alternatives”; (**c**) AB foods without milks; (**d**) number of items of all food products considering both PB (in green colour) and AB foods (in orange colour).

Figure 1 shows the amount of beverage and food items collected, overall and by food group for PB and AB foods. We included PB “meat alternatives” (134 items); PB “pate alternatives” (64 items), PB “milk alternatives” (313 items), including oat, almond, and soy “milk”, PB “yoghurt” (12 items), and PB “cheese alternatives” (21 items). AB foods were classified as whole milk (79 items), skimmed milk (50 items), semi-skimmed milk (75 items), pate (16 items), cheese (16 items), yoghurt (16 items), and meats (121 items from hamburgers and sausages). 

### 3.1. Nutrient Composition and Characteristics of PB and Animal-Based Foods

**Figure 2:**
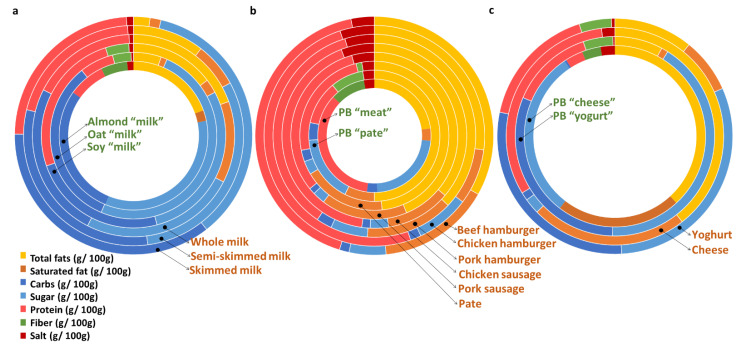
Nutrient profile of the PB (in green colour) and AB foods (in orange colour) relative to 100 g or 100 mL: (**a**) group of beverages including PB “milk alternatives” (almond, oat, and soy “milk”) and AB milk (whole, semi-skimmed, and skimmed cow milk) items; (**b**) PB “meat alternatives”, PB “pate alternatives”, and AB hamburgers and sausages (beef, chicken, or pork based); (**c**) PB and AB dairy products (yoghurt and cheese), excluding milks. Each circle represents one type of food, while the different colors of lines represent the grams of nutrient per 100 g of product, as indicated in the legend, in dry weight. The percentage range of water per food group is: (**a**) 84–91%; (**b**) 52–70.5%; (**c**) 37.09–46.44% per cheese products; and 73.01–77.39% per yoghurts.

The nutritional features of the food products included in this study are shown in Figure 2. Regarding vegetable milks (Figure 2a), i.e., PB “milk alternatives”, all contained fiber and a relatively high amount of carbs and sugar per 100 mL. Interestingly, these milks were poor in proteins, except soy “milk”, and in fats, mostly in the case of oat “milk”. Similarly, the other PB “dairy product alternatives” (Figure 2c), cheese and yoghurt, were also characterized by containing fiber, a low amount of proteins and fats (except in cheese), and a high amount of carbs (mostly in yoghurt). PB “cheese alternatives” had outstanding amounts of fats and saturated fats. As for other PB foods (Figure 2b), a similar pattern was observed: presence of fiber, but absence of saturated fats (except for PB “pate alternatives”, and to some extent, PB “milk alternatives”), and low content of proteins.

With respect to AB foods, the content in proteins appeared to be high in all types of cow milk (Figure 2a). For other AB foods (Figure 2b,c), varying nutrient compositions were observed. These foods shared the common feature of a very high protein content. Salt and saturated fats were also over-represented in these foods. Overall, AB foods lacked fiber, except in yoghurt (some brands contained fruits) and in pate (some brands contained vegetables or pulses).

### 3.2. Nutritional Comparison between PB Foods and AB Analogous Foods

#### 3.2.1. PB “Milk Alternatives” vs. AB Milks

Table 1 shows the nutrient composition of PB ”milk alternatives” and AB milks and their differences. Figures S1–S3 show the same for oat, almond, and soy “milk”, with respect to AB milks. The highest energy value per 100 mL was observed for whole milk, driven by the higher fat content of this milk (3.6 g) compared to all PB “milk alternatives” (0.9 g to 1.8 g fat, *p* < 0.001). While some PB “milk alternatives” contained fats due to the constituent of these milks (soy or almonds), the presence of saturated fats was relatively low and similar to that of skimmed cow milk. On the contrary, the total carbs and sugar content was higher in PB “milk alternatives” compared to cow milks. Particularly, oat “milk” showed the highest content of these nutrients (8.3 g and 5.5 g, respectively), with respect to almond “milk” (3 g carbs; *p* < 0.001), soy “milk” (3.5 g carbs; *p* < 0.001), and also with respect to all types of cow milks (4.6 g to 4.8 g carbs; *p* < 0.001). Sugars were notably higher in oat “milk” with respect to the other PB “milk alternatives” (2.5 g to 2.9 g; *p* < 0.001). Other findings to note are that fiber was only present in PB “milk alternatives”, and the protein content was alike in the tree types of cow milks, as well as in soy “milk” (3.1 g). However, the amount of proteins was much lower in almond “milk” (0.7 g) and oat “milk” (1.1 g) when compared with all other milks (*p* < 0.001). 

In relation to the adequacy of nutrients provided by 100 mL PB “milk alternatives” and AB milks according to the recommended intakes (Table S1 for an adult with average energy intake of 2000 kcal), soy “milk” provides 6% of the requirements for proteins, similar to that provided by cow milk. However, other types of PB “milk alternatives” provide only 1.5 to 2% of the recommendations for protein intake.

#### 3.2.2. Nutrient Composition of PB Foods

Table 2 shows the nutrient composition of PB and AB foods. Within both groups, the following results were observed:

The median energy content of all PB “meat” analogues was 177 kcal per 100 g. Among PB foods, “cheese alternatives”, and “pate alternatives”, were even more energy dense. PB “cheese alternatives” and PB “pate alternatives” had by far the highest fat content, whereas PB “yoghurt alternatives” seemed to have the lowest. Interestingly, PB “cheese alternatives” had a remarkably high content of saturated fats (14 g/100 g). Saturated fats were present in other PB foods, albeit in smaller amounts. Sugars were relatively high in PB “yoghurt alternatives”. Regarding proteins, the amount of this nutrient was not outstanding in PB foods, with an overall content of less than 5 g proteins, except in PB “meat alternatives” (14 g proteins). All PB foods were a source of fiber (1 to 9 g fiber), with PB “meat alternatives” presenting the highest amount of this component. Finally, all PB foods, except “yoghurt alternatives”, contained salt (~1 g).

Among AB foods, cheese and pate were also foods with a notably high value of calories (>250 kcal per 100 g), fats (>20 g), and saturated fats (9 to 12.5 g). The median protein content was 14 per 100 g in meats, and 7 g per 100 g cheese, while 3.8 g per 100 g yoghurt. A certain amount of fiber was present in pate and yoghurt, likely provided by some of their ingredients (vegetables/legumes and fruits, respectively). As in PB foods, all AB foods, except yoghurts, contained salt (~1 g).

#### 3.2.3. PB Foods vs. AB Foods

**Figure 3:**
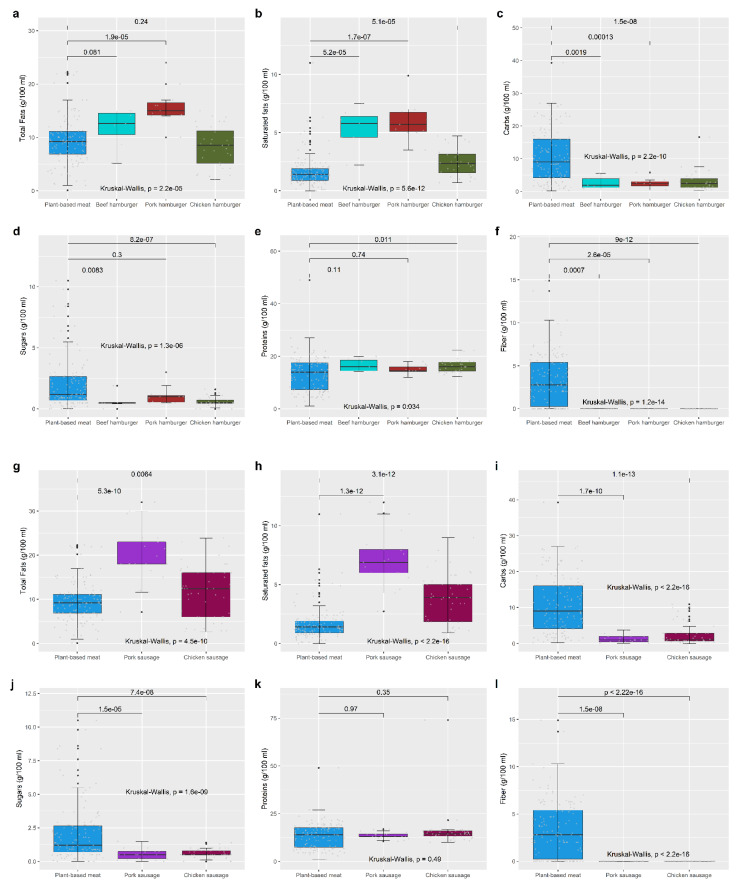
Boxplots of the nutrient composition, per 100 g of AB meats (burger and sausages) and PB alternatives. PB “meat” (blue colour) is compared with AB meat products (beef burger in light blue, pork burger in red, chicken burger in green, pork sausage in purple, and chicken sausage in magenta colour). For both hamburgers and sausages, the nutrient composition is shown for total fats (**a**,**g** respectively) and saturated fats content (**b**,**h**, respectively), carbs (**c**,**i**, respectively) and sugars content (**d**,**j**, respectively), proteins (**e**,**k**, respectively), and fiber (**f**,**l**, respectively), relative to 100 g meat. *p*-values are derived from Wilcoxon test (between two groups) and from Kruskal–Wallis tests (between more than two groups).

In Figure 3, comparisons between the PB “meat alternatives” and the AB counterparts (burgers and sausages) are shown. Figure S4 accounts for the same, but for the groups of PB “pate alternatives”, “cheese alternatives”, “yoghurt alternatives”, and “meat alternatives” compared with their AB counterparts. Table 2 also shows differences in the median nutrient content between PB and the AB analogues:

(1) PB “pate alternatives” vs. AB pate (Figure S4, Table 2): PB “pate alternatives” was significantly richer in carbs (*p* < 0.001), but not in sugars or fiber, compared to AB pate. While both types of products had a similar median content of fats, the median content of saturated fats was significantly higher in the AB pate (*p* < 0.001). The median amount of proteins also differed significantly between PB and AB pate, with the latter one showing a higher content of this nutrient (*p* < 0.001). The quantity of total salt also seemed to be higher in the AB pate compared to the PB alternative (*p* = 0.006). Both products had a similar caloric contribution.
(2) PB “cheese alternatives” vs. AB cheese (Figure S4, Table 2): The energy median value did not differ significantly between PB “cheese alternatives” and AB cheese. However, there were significant differences in the median content of proteins (higher in AB cheese, *p* < 0.001), fiber (higher in PB “cheese alternatives”, *p* = 0.03), carbs (higher in PB “cheese alternatives”, *p* < 0.001), and salt (higher in PB” cheese”, *p* = 0.03). As aforementioned, an unexpected finding was the relatively high amounts of fats and saturated facts in PB “cheese alternatives”, which were similar to those found in AB cheese.
(3) PB “yoghurt” vs. AB yoghurt (Figure S4, Table 2): Both groups showed few differences regarding the nutrient composition. Indeed, significant differences were only found regarding saturated fat, with AB yoghurts having a higher median value than PB “yoghurt alternatives” (*p* = 0.03).
(4) PB “meat” vs. AB meat (Figure 3 and Figure S4, Table 2**)**: PB “meat” appeared to provide a similar quantity of energy than the other AB meats, and nearly the same amount of proteins. However, the nutrient composition differed significantly from each other. Compared to AB meats (either beef, pork, or chicken meats), the PB “meat” alternatives were richer in total carbs and sugars (*p* < 0.001). In addition, PB “meat alternatives” had a lower median content of fats compared to pork burgers or sausages (*p* < 0.001), and a notably lower median content of saturated facts compared to all other meats, most outstanding when compared to pork meats (*p* < 0.001) and chicken sausage (*p* < 0.05). AB meats also lacked fiber.

Regarding the adequacy of nutrients provided by 100 g of PB foods compared to their AB counterparts, and with reference to the recommended intakes for adults (average energy intake of 2000 kcal), as shown in Tables S2–S4, PB foods are furthest from achieving the recommended intakes for proteins. Some exceptions are in PB “meat alternatives”, which account for a similar amount of the recommended intakes than AB meats (around 25%), and in PB “yoghurt alternatives” and AB yoghurt, accounting similarly to the daily recommended protein requirements (around 7%). The highest difference in the protein supply was seen for PB “cheese alternatives” and AB cheese (2% and 17% of protein requirements, respectively), followed by PB “pate alternatives” and AB pate (9% and 23% of protein requirements, respectively).

### 3.3. Labelling Scores: Nutri-Score, NOVA, and Eco-Score

#### 3.3.1. Nutri-Score, NOVA, and Eco-Score Classifications in PB “Milk Alternatives” and PB Foods

**Figure 4:**
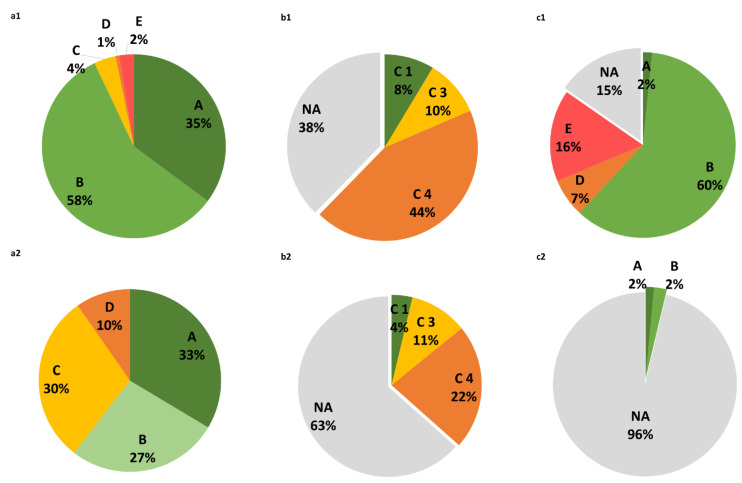
Pie charts of the distribution of foods into the categories set by Nutri-Score, NOVA, and Eco-Score. Nutri-Score (**a1**,**a2**), NOVA index (**b1**,**b2**), and ECO-score (**c1**,**c2**) of PB “milk alternatives” (**a1**,**b1**,**c1**) and PB foods (**a2**,**b2**,**c2**). The percentage of products with a certain score is indicated. NA: not available.

Figure 4 shows the categories of the different index scores of the pack labelling for PB “milk alternatives” and foods. The majority of PB “milk alternatives” (93%) were classified as A and B according to Nutri-Score, and fewer products were worse rated (7%) (Figure 4(a1)). In contrast, more than half of the other PB foods were rated as A or B, around one third of these products were assigned to C, and the remaining to D (Figure 4(a2)).

With regard to the NOVA index (Figure 4(b1,b2)), it was found that a large proportion (>35%) of both PB “milk alternatives” and other PB foods (>60%) did not have this information on the labelling. Among those products with NOVA information available, there were more than 40% of the PB “milk alternatives” classified as ultra-processed foods, compared to 22% of PB foods that were classified in this category.

Information regarding the ECO-score was given for 85% of the PB “milk alternatives” to reinforce the sustainable value of these products, but rarely provided in the other PB foods. Around 60% of the PB “milk alternatives” that had this information were indicated to have a low ecological impact, compared to less than 5% of the other PB foods.

Differences in the proportions of Nutri-Score, NOVA, and Eco-Score categories between PB “milk alternatives” and foods were all statistically significant (data not shown).

#### 3.3.2. Nutri-Score, NOVA, and Eco-Score Comparison by PB Foods and Milks, and by AB Food Analogues

As shown in greater detail in Table 3 for PB foods and Table S5 for AB foods, the distribution of the different index scores of the pack labelling also differed by food groups. Among PB foods (Table 3), “dairy product alternatives” (“milk” and “yoghurt”) had the most complete information on the index scores. Both were more frequently categorized as Nutri-Score B (over 55% of the products), and as Eco-Score B. Nevertheless, about 25% of the PB “milk alternatives” received an Eco-Score rating of D or E (high impact). It is important to point out that around 95% of PB “meat alternatives” and “pate alternatives” had no information on this score.

Nutri-Score A and B accounted for more than 50% of all PB “meat alternatives”, while Nutri-Score C, D, or E were more often present in PB “cheese alternatives” and “pate alternatives”. Missing information in NOVA was also prominent in some food groups. Nonetheless, the category 4 was more commonly assigned to PB “meat alternatives” and PB “cheese alternatives”, as well as to PB “milk” and PB “yoghurt”. For instance, 146 of the PB “dairy product alternatives” (43%), including almost all yoghurts, were rated as NOVA 4. Thus, the nutritional quality assessed by Nutri-Score and NOVA was opposite for some food groups, such as for PB “meat alternatives”: 33% PB “meat alternatives” were ranked as A in Nutri-Score, while 22% were of NOVA 4.

In relation to AB foods (Table S5), milk and yoghurt were more commonly of Nutri-Score A and B (80 and 50%, respectively), whereas cheese, meat, and pate were of Nutri-Score C, D, and E. In NOVA, for which more than half of meats and milks lacked this information, the majority of pates and yoghurts were of category 4. Remarkably, 25% the cheese products also fell into this category. The nutritional quality for AB meats assessed by Nutri-Score (70% ranked as C or D) was in line with the processing degree assessed by NOVA (27% ranked as 4). Information on the Eco-score was quite complete, the majority (except yoghurt) being classified as D and E (high ecological impact).

### 3.4. Ingredients and Additives Used in PB “Milk Alternatives” and Foods

Common additives (% of use) among PB “milk alternatives” were emulsifiers and stabilizers including lecithin from soybeans or sunflower seeds (11%), citric acid esters of mono- and diglycerides of fatty acids (2%), gellant gum (33%), guar gum (3%), and locust vegan gum (4%). Of note, 7% of the PB “milk alternatives” contained red algae extracts as a source of calcium. In addition, PB “milk alternatives” had added calcium or potassium through calcium orthophosphates (13%), tricalcium phosphate (12%), sodium or potassium citrate (1%), mono or dipotassium phosphate (2%), and calcium carbonate (9%).

In PB “meat alternatives” and PB “pate alternatives”, the most frequently used ingredient was soy (in 55% of these food products), followed by wheat or wheat gluten (14%) and eggs (6%). Additives used in these products were thickeners and stabilizers such as methyl cellulose (13%), xanthan gum (14%), and other gums. In PB “cheese alternatives”, it was found that 52% of this foodstuff contained coconut oil as a main ingredient. Starch was another important ingredient in 67% of the products. Modified starches were also used as a main additive in this food product. Focusing on PB “meat alternatives”, the common ingredients were soy (in 72% of these products), wheat (22%), pea and chickpea flour (20%), and eggs (8%). Regarding PB “pate alternatives”, the most frequently used fats as ingredients were sunflower oil (61%), one of which was high oleic, olive oil (28%), and extra virgin oil (20%). There were few PB “pates” (N = 3) that contained palm or canola oil.

### 3.5. Nutrition and Health Claims Used in PB “Milk Alternatives” and PB Foods

Nutrition claims are summarized as counts and percentages in Table 4. The following nutrient fortifications (% of foods with a claim by food groups) regarding minerals and vitamins were found in PB “milk alternatives” and PB foods: calcium (15%) and vitamins (13%)—mainly E, B2, B12, A, and D vitamins in almond “milk”; B2, B12, and D vitamins in oat “milk”; and B2, B12, A, and D in soy “milk”.

Concerning nutrition claims (% of foods with a claim by food groups) according to Regulation (EU) 1169/2011, some PB foods and PB “milk alternatives” were indicated to be sources of calcium (34% of “milk alternatives”), sources of proteins (9% of “milk alternatives” and 21% of other PB foods), sources of vitamins (24% in “milk alternatives” and 0.4% in other PB foods), sources of fiber (2% of “milk alternatives” and 16% of other PB foods), sources of iron (1% of all PB foods), low sugar (7% of “milk alternatives”), low fat (9% of “milk alternatives” and 5% of other PB foods), and low sodium (3% of “milk alternatives”).

Apart from nutrition claims, other statements in PB foods were related to the non-presence of ingredients or allergens such as additives (0.7%), gluten (15%), lactose (11%), or soy (1%). Additionally, 21% of these foods had an organic declaration, and some (0.7%) were labelled as GMO free foods. There were no health claims reported in these products.

## 4. Discussion

This study presents a comprehensive overview of the nutritional characteristics and nutrient profiling models provided in the pack labelling of 544 vegetarian foods (meat and dairy alternatives) marketed in Spain. Several differences in the nutritional composition of these foods have been encountered. In general, these food products were mostly ranked in Nutri-Score categories A and B; at the same time, some of them belonged to NOVA categories 3 and 4. In comparison to some homologous AB food groups, notable variations in the nutritional quality and in the index scores classifications were observed. The largest gaps were, in essence, that PB foods seemed to feature a healthier nutrient composition in terms of fats, saturated fats, and fiber, but PB alternatives could also be inappropriate to provide proteins at the same levels as meat and dairy products. The high content of carbs and sugars in some PB food compared to the AB counterparts was also noteworthy. Some exceptions were observed regarding PB “cheese alternatives” and PB “pate alternatives” (high-fat products), as well as PB “meat alternatives” (high-protein products).

PB food products have emerged in recent years to suit the needs of health-conscious consumers or vegetarians, who are demanding more sustainable and healthier foods [2]. This is why many new PB products, such as meat and dairy product alternatives, that mimic the organoleptic characteristics of AB foods, have come into the market [48]. The current study included a study sample of 544 PB foods, including 134 PB “meat” analogues, 313 PB “milk alternatives” and 97 other PB foods (“pate”, “cheese”, and “yoghurt”), as well as 373 AB foods for comparison purposes. Two previous European studies have retrieved information on PB “meat products” marketed in Italy (*N* = 269 PB “meat” analogues) [31], and the UK (*N* = 207 PB “meat” analogues, including burgers and meatballs) [32]. A German study also evaluated the nutritional characteristics of 316 PB “meat alternatives” and 159 PB “cheese alternatives” alternatives [33]. Beyond Europe, other studies have also attempted to evaluate the nutritional features of PB “meat alternatives” (e.g., 137 PB “meat alternatives” in Australia, and 37 PB beef alternative products in the US) [49,50], but comparisons with the AB counterparts were not undertaken in these studies. PB beverages as milk substitutes and PB “dairy products” have also received attention, as evidenced in some studies carried out in the UK (*N* = 136 PB “milk alternatives”, 55 PB “yoghurt alternatives”, and 109 PB “cheese alternatives”) [51], and in other countries [52,53]. However, other types of PB foods were not included in these previous studies. A summary of the data reported in these studies is provided in Table S6.

The protein content of PB “meat alternatives” in our study (14 g/100 g, on average) was close to that of the corresponding AB meat products, which is in agreement with results reported in other studies (~16 g/100 g for burgers and meatballs) [31,32]. We could not confirm previous findings regarding differences by type of meat [32], where plant-based steaks were found to have the best nutritional characteristics. Indeed, in our study, the majority of PB “meat alternatives” were “burgers” and “sausages” made of similar ingredients. By contrast, we considered different types of AB meat products (sausages, burgers, and red and white meats) for the comparative study. Overall, PB “meat alternatives” showed a more favorable nutrient composition than AB meats. Regarding essential amino acids, as has been reported before [54,55], their amount should be lower in PB “meat alternatives” (for example, soy protein isolates reach 27% of the recommended content) compared to AB meats, where all essential amino acids are provided. However, we did not examine the food´s content of these amino acids. In addition, PB “meat alternatives” are of a meaty texture, appearance, and meat-like flavour. To achieve this goal, specific additives, processes of fractioning of whole foods into substances, and chemical modifications of these substances are required [29,56]. The supplement of protein in these foods can be also fulfilled by incorporating protein-rich vegetable materials and by adopting a proper technological process [54,57]. The vegetable base of these PB “meat alternatives” are often cereals and pulses, as they can easily enhance the nutritional and functional features of these products [58]. Our study reveals that traditional protein sources are being used in Spanish PB foods.

PB “milk alternatives” that were included in our study also had a varied nutritional composition. For instance, the protein content of almond “milk” was the lowest (<1 g/100 mL), whereas that of soy “milk” was the highest (>3 g/100 mL). Protein levels of soy “milk” were even comparable to that of cow milk. Similar results have been obtained in previous studies on this issue [51,52,59]. Nevertheless, the protein quality and content of essential amino acids in PB “milk alternatives” is lower than that of cow milk [54]. A key finding that has also been underscored in other studies is related to oat “milk” [53,59]. Oat “milk” presented a nutrient composition characterized by a low protein content compared to the other PB “milk alternatives” and the AB counterparts, as has been also reported by others [53]. While it was not possible to analyze further the nutritional composition of the PB “milk alternatives”, other studies have reported that calcium content of oat “milk” is similar to that of cow milk [59]. However, its bioavailability should be also taken into account, since PB foods contain compounds that could limit calcium absorption. In general, cow milk contains more energy, saturated fat, protein, and also vitamin B2, vitamin B12 and iodine, than PB “milk alternatives” [52].

For the other PB foods, we also found some striking differences when comparing the nutrient composition with the AB counterparts. PB “pate alternatives”, for example, showed a lower nutritional quality given its high fat content and low protein supply. The same was observed for PB “cheese alternatives”, which showed a high value of both fats and saturated fats. PB “cheese alternatives” are made of coconut oil and other eatable oils of industrial use, which might explain these findings. PB “pate alternatives” contained other vegetables oils (mostly olive oil) in our study samples. The same has been observed for PB “cheese alternatives” in the study on PB foods on the German market [33]. The nutrient composition of PB “yoghurt alternatives” resembled that of the AB counterparts regarding proteins and other nutrients, although providing less saturated fats and more fiber, as has been also observed in other studies [51].

Regarding food classification systems on the food labels, it is important to note that half of the PB foods in Open Food Facts did not have information on the NOVA score available, and only PB “milk alternatives” provided the ECO-Score label. Nutri-Score only accounts for the nutritional quality of food products, but does not consider the degree of the processing, as has been shown in a study comparing both labelling systems on Open Food Facts data [38]. Likewise, in our study, while most of the PB foods included were ranked as Nutri-Score A and B (good nutritional quality), some were further classified as NOVA 3 and 4 (processed and ultra-processed, respectively). This was the case of PB “meat”, for example. A call to pay attention to the need to incorporate this information should be made to guide consumer’s choices of the healthier and less processed PB foods on the market. Several studies have even requested that Nutri-Score should be accompanied by the NOVA category to provide this information [38,60]. At the same time, efforts should be made to enhance people´s understanding of these food label systems, since this knowledge is limited in some sectors of the population [61].

While PB dietary patterns are characterized by avoiding the intake of meat and meat products, different PB dietary patterns exist. The strictest PB dietary pattern is the vegan diet, which is characterized by excluding all kinds of foods of animal origin (meat, fish, seafood, dairy, and eggs). The ovo-lacto-vegetarian diet allows the consumption of eggs and dairy products; the pesco-vegetarian diet includes fish, seafood, dairy products, and eggs; and a flexitarian diet is featured by consuming mainly PB foods, but allows the consumption of meat and fish occasionally [28]. All, but particularly vegans, are prone to nutritional deficiencies (iron, vitamin B12, and others) [10,11,12]. In fact, meat and dairy products are the major providers of proteins, vitamins, and minerals in the diet, but PB “meat alternatives” and “milk alternatives” lack the nutrients contained in these foods [51]. As our study shows, in general, proteins contained in PB foods cover a lower percentage of the reference intakes for adults than those provided by AB foods. However, as a recent systematic review of 141 observational and intervention studies showed, the protein intake in a vegan or vegetarian diet still meets the recommended intake levels [62]. Therefore, PB diets are being promoted as a healthy dietary pattern, considering that food fortification and dietary supplements can alleviate the nutritional shortcomings of this dietary pattern. Our study also shows that a number of PB foods, mostly PB “milk alternatives”, were fortified with some of these nutrients. However, nutrition claims were given only in some cases (in less than 15% of all PB foods).

As mentioned before, current public health nutrition policies are promoting the consumption of nutrient-dense PB foods in the population due to the healthiness of PB diets [63]. Indeed, PB foods are considered a protective factor against the development of cancer [19,64], and of cardiovascular diseases (CVD) [63]. For instance, the intake of vegetable-source monounsaturated and polyunsaturated fatty acids (mostly linoleic and a-linoleic acids) are associated with an improved CVD risk profile [13]. However, PB foods can also contain refined starches and simple sugars, which have been associated with the opposite effects [18]. A distinction is therefore made between the healthy and the unhealthy PB dietary patterns [65,66]. In this regard, there is also a need to evaluate the nutritional composition and quality of PB foods on the market. The results of our study show that the nutritional composition varies greatly between the PB foods, with PB “cheese alternatives” being the most energy-dense food and PB “milk alternatives” being the lowest. As aforementioned, PB “cheese alternatives” also contained an unexpectedly high amount of saturated fats, whereas PB “yoghurt alternatives” were low in this nutrient.

The nutritional information on AB foods was taken from Open Food Facts and other online resources to follow the same procedure applied to retrieve information of PB foods, and to get the information on the nutrient profile index scores and ingredients of the food labels. In addition, given that some food products were varied (for example, sliced cheese or cream cheese, Greek-style yoghurt, or fruit yoghurt), we aimed to include a sample of each. While it is possible that this issue could pose a problem when comparing these features with regard to the PB foods, we took into consideration those AB products that appeared to be more similar to the PB ones. Regarding PB “meat alternatives” and PB “milk/dairy alternatives”, special care was taken to collect AB products of different nutritional compositions, for example, by accounting for whole and skimmed milks, as well as red and white meats (from high to low fat content, etc.). In addition to the exhaustiveness of our study, another strength to highlight is the fact that we evaluated the nutrient composition of different PB foods (five food groups), with each other and in comparison with some of their AB counterparts, as well as the nutritional quality, the processing degree, and the ecological impact, together with the ingredients, and the nutritional and health claims reported on the food labels. A limitation to note is that we could not consider nutrients different to those reported on these labels, such as vitamins and minerals, except when these nutrients were part of the nutritional claim or when they were added. Moreover, the NOVA and ECO-Score were available for only some of the food products in Open Food Facts [37]. Furthermore, we cannot guarantee the reliability of the nutritional information of the labels since no chemical analyses were undertaken in this study. In addition, we selected PB foods (meat and dairy alternatives) available on the Spanish market, but different PB food formulations with other nutrient profiles and compositions may exist in other countries; this fact could jeopardize the extrapolation of our results.

## 5. Conclusions

PB alternative products of meat, milk, and dairy products marketed in Spain are numerous and diverse, and seem to provide varied amounts of nutrients, including proteins, carbs, sugars, fats, and saturated fats. While all are rich in dietary fiber and vegetable proteins, compared to their AB counterparts, these foods do not usually provide higher protein rates than AB foods. The nutritional composition of PB “milk alternatives” also varies greatly depending upon the base vegetable that they are made from. Even if most of these PB foods typically have a low Nutri-Score, some can be regarded as ultra-processed due to the high degree of processing of these foods. However, it should also be noted that PB foods often do not provide information on the processing or ecological impact on the food label, despite their relevance. Thus, since PB foods may not always represent a healthier alternative to an AB product, it is important to advise consumers to pay attention to the nutrition labelling of these products. Commercial PB foods should also have specific denominations since they are quite different to the AB foods that they try to mimic regarding ingredients and technological processes used in their formulation. Furthermore, it is highly necessary to conduct more studies on vegetarian-like foods that are becoming available on the market in order to gain a deeper understanding about the differences between PB and AB foods regarding nutritional quality, ingredients, and processing. This information would also aid in new product development, which should aim for a minimum protein content, a maximum content of critical nutrients (fat, sugars, and sodium), a consistent nutrient fortification, and complete labelling information.

## Figures and Tables

**Table 1:** Nutrient composition from the front-of-pack labelling by beverages, per 100 mL of AB milks and PB alternatives.

	Number of Items	Energy (kcal)	Total Fat (g)	Saturated (g)	Carbs (g)	Sugars (g)	Protein (g)	Fiber (g)	Salt (g)
Almond “milk”	*N* = 80	29.0 ^ab^ [23.0;45.5]	1.70 ^ab^ [1.20;2.20]	0.20 ^ab^ [0.10;0.30]	3.00 ^a^ [0.30;5.20]	2.50 ^abcd^ [0.10;4.10]	0.70 ^acde^ [0.50;0.90]	0.50 [0.10;0.70]	0.12 [0.09;0.14]
Oat “milk”	*N* = 114	48.0 ^ac^ [45.0;53.0]	0.90 ^c^ [0.80;1.48]	0.20 ^cg^ [0.10;0.20]	8.30 ^abcde^ [7.70;9.15]	5.50 ^ae^ [4.50;6.45]	1.10 ^bfgh^ [0.80;1.40]	0.60 [0.50;0.88]	0.08 [0.06;0.11]
Soy “milk”	*N* = 119	42.0 [34.8;47.2]	1.80 ^de^ [1.60;2.00]	0.30 ^de^ [0.30;0.30]	3.50 ^b^ [1.60;4.60]	2.90 ^e^ [1.20;4.00]	3.10 ^ab^ [3.00;3.60]	0.50 [0.30;0.67]	0.10 [0.06;0.13]
Whole milk	*N* = 79	63.0 ^bd^ [63.0;63.0]	3.60 ^bcdf^ [3.60;3.60]	2.40 ^bcdf^ [2.30;2.50]	4.60 ^c^ [4.60;4.70]	4.60 ^b^ [4.60;4.70]	3.10 ^cf^ [3.00;3.10]	0 [0;0]	0.13 [0.10;0.13]
Semi-skimmed milk	*N* = 75	46.0 [45.0;46.0]	1.60 ^a^ [1.55;1.60]	1.00 ^aeg^ [1.00;1.10]	4.70 ^d^ [4.70;4.80]	4.70 ^c^ [4.70;4.80]	3.10 ^dg^ [3.10;3.20]	0 [0;0]	0.13 [0.10;0.13]
Skimmed milk	*N* = 50	35.0 ^cd^ [34.0;37.0]	0.30 ^ef^ [0.30;0.50]	0.20 ^f^ [0.19;0.20]	4.80 ^e^ [4.76;4.80]	4.80 ^d^ [4.76;4.80]	3.20 ^eh^ [3.15;3.20]	0 [0;0]	0.13 [0.11;0.13]
*p*-value		<0.001	<0.001	<0.001	<0.001	<0.001	<0.001	0.001	<0.001

Median values and the interquartile range are indicated [p25–p75]. Post hoc Tuckey test was applied for pair-wise comparisons between groups by the nutrient composition. *p*-values < 0.001 in these pair-wise comparisons are indicated in letters (from a to h). *p*-values derived from non-parametric tests for median comparisons: Kruskal–Wallis test (more than two groups) or Wilcoxon rank sum test (two groups) are also shown in Figures S1–S3.

**Table 2:** Nutrient composition from the pack labelling by foods, per 100 g of AB foods and PB alternatives.

	Number of Products	Energy (kcal)	Total Fat (g)	Saturated (g)	Carbs (g)	Sugars (g)	Protein (g)	Fiber (g)	Salt (g)
Meat products									
Beef hamburger	*N* = 7	198 [184;216]	12.60 ^l^ [10.50;14.60]	5.80 ^j^ [4.60;6.40]	1.90 ^e^ [1.20;3.90]	0.50 ^d^ [0.44;0.50]	16.00 ^a^ [14.50;18.50]	0 ^a^ [0;0]	1.20 [1.01;1.49]
Chicken hamburger	*N* = 32	158 ^a^ [130;184]	8.55 ^abcj^ [5.18;11.20]	2.35 ^abi^ [1.55;3.15]	2.50 ^a^ [1.20;3.92]	0.50 ^c^ [0.45;0.72]	16.00 ^b^ [14.40;17.80]	0 ^b^ [0;0]	1.63 [1.40;1.90]
Pork hamburger	*N* = 11	208 [198;220]	15.00 [14.20;16.50]	5.70 ^f^ [5.11;6.75]	2.40 ^f^ [1.65;3.00]	1.00 [0.55;1.10]	14.50 ^e^ [14.20;16.00]	0 ^c^ [0;0]	1.86 [1.48;2.10]
Chicken sausage	*N* = 50	180 ^bd^ [130;202]	12.40 ^defk^ [6.00;16.00]	3.90 ^ck^ [1.85;5.00]	1.10 ^b^ [0.76;2.90]	0.50 ^b^ [0.50;0.80]	14.40 ^c^ [13.30;16.00]	0 ^d^ [0;0]	2.00 ^ab^ [1.72;2.20]
Pork sausage	*N* = 21	225 ^d^ [219;262]	18.00 ^cfi^ [18.00;23.00]	6.90 ^bgh^ [6.00;8.00]	1.00 ^c^ [0.50;2.00]	0.50 ^a^ [0.20;0.75]	13.00 ^f^ [13.00;14.40]	0 ^e^ [0;0]	1.80 [1.70;2.00]
Pate	*N* = 16	269 [254;277]	23.20 ^adg^ [20.20;24.30]	8.70 ^acde^ [7.84;9.10]	1.35 ^d^ [1.28;5.12]	1.15 [1.00;1.30]	11.60 ^g^ [10.4;11.9]	0.55 [0.28;0.83]	1.80 [1.50;1.90]
PB “meat”	*N* = 134	177 ^ce^ [159;212]	9.20 ^ghijkl^ [6.88;11.10]	1.40 ^dfgijk^ [0.90;1.90]	9.05 ^abcef^ [4.15;16.00]	1.20 ^abcd^ [0.70;2.65]	14.00 ^d^ [7.35;17.6]	4.00 ^abcde^ [2.55;6.18]	1.20 ^a^ [0.81;1.60]
PB “pate”	*N* = 64	264 ^abc^ [196;328]	20.90 ^beh^ [15.90;31.00]	3.05 ^eh^ [2.00;4.80]	6.90 ^d^ [4.38;8.93]	1.75 [0.50;4.12]	4.50 ^abcdefg^ [2.08;6.53]	3.36 [2.00;4.72]	1.19 ^b^ [1.00;1.60]
*p*-value		<0.001	<0.001	<0.001	<0.001	<0.001	<0.001	<0.001	<0.001
Dairy products									
Cheese	*N* = 16	272 ^ab^ [219;352]	21.10 ^abc^ [16.20;28.50]	12.50 ^abc^ [11.30;17.4]	1.25 ^ab^ [0.50;2.03]	0.65 ^a^ [0.40;2.03]	17.00 ^abcd^ [12.30;25.00]	0.00 ^a^ [0.00;0.07]	1.06 ^abc^ [0.78;1.60]
Yoghurt	*N* = 16	75.0 ^be^ [57.8;96.0]	2.45 ^cf^ [1.55;3.23]	1.70 ^cf^ [0.85;2.20]	6.95 ^e^ [4.60;12.20]	6.60 ^c^ [4.27;12.10]	3.80 ^a^ [3.30;4.65]	1.0 [1.00;1.00]	0.11 ^ae^ [0.10;0.12]
PB “cheese”	*N* = 21	311 ^c^ [281;362]	24.00 ^def^ [22.50;28.80]	14.00 ^def^ [5.20;20.00]	19.00 ^ac^ [11.00;23.00]	0.21 ^bc^ [0.00;2.98]	2.00 ^b^ [0.50;14.20]	2.10 [0.50;8.10]	1.60 ^def^ [1.20;2.20]
PB “yoghurt”	*N* = 12	79.5 ^acd^ [73.5;84.2]	2.10 ^be^ [1.98;2.15]	0.30 ^be^ [0.30;0.40]	11.20 ^d^ [9.55;13.50]	8.35 [5.45;10.30]	3.65 ^c^ [2.58;3.70]	1.30 ^b^ [0.30;1.40]	0.09 ^bf^ [0.07;0.14]
*p*-value		<0.001	<0.001	<0.001	<0.001	<0.001	<0.001	<0.001	<0.001

Median values and the interquartile range are indicated [p25–p75]. Post hoc Tuckey test was applied for pair-wise comparisons between foods groups (meats, dairy products, and others) by their nutrient composition. *p*-values < 0.001 in these pair-wise comparisons are indicated in letters (from a to l). *p*-values derived from non-parametric tests for median comparisons: Kruskal–Wallis test (more than two groups) or Wilcoxon rank sum test (two groups) are also shown in Figure 4 and Figure S4.

**Table 3:** Nutrient profiling models according to Nutri-Score, NOVA, and ECO-Score for PB “milk alternatives” and other PB foods.

	PB “Milk”	PB “Meat”	PB “Pate”	PB “Yoghurt”	PB “Cheese”
	*N* = 313	*N* = 134	*N* = 64	*N* = 12	*N* = 21
NUTRI-SCORE					
A	110 (35.14%)	45 (33.58%)	6 (9.23%)	4 (33.33%)	0 (0%)
B	181 (57.83%)	36 (26.87%)	11 (16.92%)	7 (58.33%)	0 (0%)
C	12 (3.83%)	40 (29.85%)	21 (32.31%)	1 (8.33%)	4 (19.05%)
D	2 (0.64%)	13 (9.70%)	23 (35.38%)	0 (0%)	6 (28.57%)
E	8 (2.56%)	0 (0%)	3 (4.62%)	0 (0%)	11 (52.38%)
Missing	0 (0%)	0 (0%)	0 (0%)	0 (0%)	0 (0%)
NOVA					
1	27 (8.63%)	5 (3.73%)	1 (1.54%)	1 (8.33%)	0 (0%)
2	0 (0%)	0 (0%)	0 (0%)	0 (0%)	0 (0%)
3	31 (9.9%)	14 (10.45%)	38 (58.46%)	0 (0%)	3 (14.29%)
4	137 (43.77%)	30 (22.39%)	12 (18.46%)	9 (75%)	8 (38.10%)
Missing	118 (37.70%)	85 (63.43%)	13 (20%)	2 (16.67%)	10 (47.62%)
ECO-SCORE					
A	5 (1.60%)	2 (1.49%)	0 (0%)	0 (0%)	0 (0%)
B	189 (60.38%)	3 (2.24%)	2 (3.08%)	9 (75.00%)	0 (0%)
C	0 (0%)	0 (0%)	0 (0%)	0 (0%)	0 (0%)
D	21 (6.71%)	0 (0%)	0 (0%)	0 (0%)	3 (14.29%)
E	50 (15.97%)	0 (0%)	0 (0%)	0 (0%)	0 (0%)
Missing	48 (15.34%)	129 (96.27%)	62 (95.38%)	3 (25%)	18 (85.71%)

Number of foods with Nutri-Score, NOVA, and Eco-Score levels; the percentage of food group per level is indicated between brackets.

**Table 4:** Nutrition claims, and other information related to ingredients and others, reported in the pack labelling of PB food products.

	Almond “Milk”	Oat “Milk”	Soy “Milk”	PB “Milks”	PB Foods	Total PB Foods and “Milks”
	*N* = 80	*N* = 114	*N* = 119	*N* = 313	*N* = 231	*N* = 544
Information relative to nutritional claim						
Low salt/sodium	4 (4.94%)	4 (3.51%)	2 (1.68%)	10 (3.30%)	0 (0%)	10 (1.84%)
Low fat/low saturated fat	9 (11.11%)	11 (9.65%)	8 (6.72%)	28 (9.24%)	12 (5.19%)	40 (7.35%)
Low sugar	0 (0%)	(0%)	21 (17.65%)	21 (6.93%)	0 (0%)	21 (3.86%)
Without added sugar ^1^	29 (35.80%)	38 (33.33%)	2 (1.68%)	69 (22.77%)	2 (0.87%)	71 (13.05%)
Source of/rich in vitamins	17 (20.99%)	14 (12.28%)	42 (35.29%)	73 (24.09%)	1 (0.43%)	74 (13.6%)
Source of/rich in calcium	21 (25.93%)	27 (23.68%)	54 (45.38%)	102 (33.66%)	0 (0%)	102 (18.75%)
Source of/rich in iron	0 (0%)	1 (0.88%)	2 (1.68%)	3 (0.99%)	3 (1.3%)	6 (1.1%)
Source of/rich in fiber	0 (0%)	6 (5.26%)	0 (0%)	6 (1.98%)	36 (15.58%)	42 (7.72%)
Source of/rich in protein	0 (0%)	0 (0%)	28 (23.53%)	28 (9.24%)	48 (20.78%)	76 (13.97%)
Information relative to ingredients						
Preservatives free	0 (0%)	1 (0.88%)	2 (1.68%)	3 (0.99%)	7 (3.03%)	10 (1.84%)
Dye free	1 (1.23%)	3 (2.63%)	3 (2.52%)	7 (2.31%)	0 (0%)	7 (1.29%)
Additives free	0 (0%)	2 (1.75%)	2 (1.68%)	4 (1.32%)	0 (0%)	4 (0.74%)
Gluten free	24 (29.63%)	6 (5.26%)	47 (39.50%)	77 (25.41%)	6 (2.6%)	83 (15.26%)
Lactose free	11 (13.58%)	13 (11.4%)	33 (27.73%)	57 (18.81%)	5 (2.16%)	62 (11.4%)
Soy free	0 (0%)	1 (0.88%)	2 (1.68%)	3 (0.99%)	2 (0.87%)	5 (0.92%)
GMO free	0 (0%)	0 (0%)	4 (3.36%)	4 (1.32%)	0 (0%)	4 (0.74%)
Information relative to environment or agriculture type						
Organic or ecological certified	20 (24.69%)	30 (26.32%)	44 (36.97%)	96 (31.68%)	18 (7.79%)	114 (20.96%)
FSC certified	12 (14.81%)	8 (7.02%)	34 (28.57%)	54 (17.82%)	(0%)	54 (9.93%)

Number of foods with nutritional claim or other information and the percentage of food group per nutritional claim or other information is indicated between brackets. ^1^ The label “with no added sugars”, according to European legislation (CE No 1924/2006), is a nutritional claim; it can be considered an ingredient information, too. This claim is also followed by the claim “contains naturally occurring sugars”. GMO: Genetically Modified Organism; FSC: Forest Stewardship Council (on forest management and chain custody).

## Data Availability

The datasets generated for this study are available on request to the corresponding author.

## Supplementary Materials

The following supporting information can be downloaded at: https://www.mdpi.com/article/10.3390/foods12061151/s1, Table S1: Coverage of daily intake (%) for nutrients by energy and selected nutrients in adults per 100 mL of PB “milks alternatives” and AB milks according to Reference Intakes; Table S2: Coverage of daily intake (%) for nutrients by energy and selected nutrients in adults per 100 mL of PB “meat alternatives” and AB homologues according to Reference Intakes; Table S3: Coverage of daily intake (%) for nutrients by energy and selected nutrients in adults per 100 mL of PB “pate alternatives” and AB pate according to Reference Intakes; Table S4: Coverage of daily intake (%) for nutrients by energy and selected nutrients in adults per 100 mL of PB “dairy product alternatives” and AB dairy products according to Reference Intakes; Table S5: Nutrient profiling models according to Nutri-Score, NOVA, and ECO-Score for AB milks and foods; Table S6: Nutritional information from the pack labelling, per 100 g of PB products reported in other studies; Figure S1. Boxplots of the nutrient composition, per 100 mL of PB oat “milk” and AB milks; Figure S2. Boxplots of the nutrient composition, per 100 mL of PB almond “milk” and AB milks; Figure S3. Boxplots of the nutrient composition, per 100 mL of PB soy “milk” and AB milks; Figure S4: Boxplots of the nutrient composition profiles, per 100 g of PB and AB foods.

## Appendix A. Description of Products, Plant- and Animal-Based, Included in the Study

**Group**	**Number of Products**	**Group Definition**
Almond “milk”	*N* = 80	Plant-based milk with watery texture manufactured from organic or non-organic almonds with or without sugar, with or without additional flavouring (coffee, chocolate, vanilla), and with or without vitamins and minerals added (calcium, vitamin A, D, E).
Oat “milk”	*N* = 114	Plant-based milk with watery texture manufactured from organic or non-organic oat with or without sugar, with or without additional flavouring (coffee, chocolate, vanilla), and with or without vitamins and minerals added (calcium, vitamin A, D2, E, B2, B6, B9, B12). This milk was also available as dehydrated oat “milk”.
Soy “milk”	*N* = 119	Plant-based milk with watery texture manufactured from organic or non-organic soybeans with or without sugar, with or without additional flavouring (coffee, chocolate, vanilla, caramel), and with or without vitamins and minerals added (calcium, vitamin A, D, B2, B9, B12).
Whole milk	*N* = 79	U.H.T. (Ultra High Temperature)or fresh whole cow milk with or without lactose from organic or non-organic farms, with or without vitamins and minerals added.
Semi-skimmed milk	*N* = 75	U.H.T. or fresh semi-skimmed cow milk with or without lactose from organic or non-organic farms, with or without vitamins and minerals added.
Skimmed milk	*N* = 50	U.H.T. or fresh skimmed cow milk with or without lactose from organic or non-organic farms, with or without vitamins and minerals added.
Beef hamburger	*N* = 7	A round cake of raw minced beef with or without additives.
Chicken hamburger	*N* = 32	A round cake of raw minced chicken with or without additives.
Pork hamburger	*N* = 11	A round cake of raw minced pork with or without additives.
Chicken sausage	*N* = 50	A chicken and/or turkey sausage manufactured from spices, minced meat, and animal fat, with or without other additives (antioxidants, emulsifiers), which are found fresh, cured, or boiled.
Pork sausage	*N* = 21	A pork sausage manufactured from spices, minced meat, and animal fat, with or without other additives (antioxidants, emulsifiers), fresh, cured or boiled.
Pate	*N* = 16	A meat paste with high fat content made with seasoned pork liver or poultry liver.
PB “meat”	*N* = 134	A prepared food specially designed and created to look like, taste like, and cook like conventional meat, hamburgers, and sausages. These products are manufactured by soy (tofu, texturized soy, soybean, tempeh), wheat (gluten), other legumes and cereals, and occasionally albumin egg.
PB “pate alternatives”	*N* = 64	A plant-based paste made with seasoned vegetables, olives, legumes, nuts, tofu, and texturized soy.
Cheese	*N* = 16	This group includes a pull of different cheese products: fresh cheese, semi-cured cheese, cured cheese, and cheese spread.
Yoghurt	*N* = 16	This group includes a pull of different fermented dairy products manufactured by whole, semi-skimmed, or skimmed milk, with or without cream, sugar, and fruit.
PB “cheese alternatives”	*N* = 21	A product made from a variety of non-dairy and non-animal products, such as nuts, other seeds, legumes, and highly in oil, mainly coconut oil.
PB “yoghurt alternatives”	*N* = 12	This group includes a pull of different fermented or non-fermented plant-based dairy products manufactured by oat or soy, with or without sugar, flavours, and fruit.
