# Supplementary material for: Characterizing Meat- and Milk/Dairy-like Vegetarian Foods and Their Counterparts Based on Nutrient Profiling and Food Labels
Source: Foods. 2023 Mar 9;12(6):1151. doi: 10.3390/foods12061151 (PMC10048389; doi:10.3390/foods12061151)
Supplement: Supplementary file 1 [file foods-12-01151-s001.zip › foods-2182309-supplementary.pdf]

foods MDPI

Supplementary Materials:

Table S1. Coverage of daily intake (%) for nutrients by energy and selected nutrients in adults per 100 ml of PB "milks alternatives" and AB milks according to Reference Intakes.

	Almond milk	Oat milk	Soya milk	Whole milk	Semi-skimmed milk	Skimmed milk
	N=80	N=114	N=119	N=79	N=75	N=50
Energy	1.45 [1.15;2.20]	2.40 [2.25;2.60]	2.10 [1.73;2.33]	3.15 [3.15;3.15]	2.30 [2.25;2.30]	1.75 [1.70;1.85]
Fats	2.43 [1.71;3.14]	1.29 [1.14;2.00]	2.57 [2.43;2.86]	5.14 [5.14;5.14]	2.29 [2.21;2.29]	0.43 [0.43;0.71]
Saturated fats	1.00 [0.50;1.50]	1.00 [0.50;1.00]	1.50 [1.50;1.50]	12.0 [11.5;12.5]	5.00 [5.00;5.50]	1.00 [0.96;1.00]
Carbs	1.04 [0.12;1.96]	3.19 [2.94;3.50]	1.35 [0.62;1.62]	1.77 [1.77;1.81]	1.81 [1.81;1.85]	1.85 [1.83;1.85]
Sugar	2.50 [0.11;4.47]	6.11 [5.00;7.00]	3.11 [0.89;3.78]	5.11 [5.11;5.22]	5.22 [5.22;5.33]	5.33 [5.29;5.33]
Protein	1.40 [1.00;1.80]	2.00 [1.60;2.60]	6.20 [6.10;7.20]	6.20 [6.00;6.20]	6.20 [6.20;6.40]	6.40 [6.30;6.40]
Salt	2.00 [1.33;2.17]	1.33 [1.00;1.67]	1.67 [0.88;2.00]	2.17 [1.67;2.17]	2.17 [1.67;2.17]	2.17 [1.83;2.17]

Median coverage [IQR: p25;p75] of reference intakes from front-of-the pack labelling (reference intakes for adults; 2,000 kcal) of plant-based milks and cow milks.

Table S2. Coverage of daily intake (%) for nutrients by energy and selected nutrients in adults per 100 ml of PB "meat alternatives" and AB homologues according to Reference Intakes.

	Plant-based meat	Beef hamburger	Chicken hamburger	Pork hamburger	Chicken sausage	Pork sausage
	N=134	N=7	N=32	N=11	N=50	N=21
Energy	8.85 [7.95;10.6]	9.90 [9.22;10.8]	7.90 [6.50;9.20]	10.4 [9.92;11.0]	9.00 [6.50;10.1]	11.2 [10.9;13.1]
Fats	13.1 [9.82;15.9]	18.0 [15.0;20.8]	12.2 [7.39;16.1]	21.4 [20.3;23.6]	17.7 [8.57;22.9]	25.7 [25.7;32.9]
Saturated fats	7.00 [4.50;9.50]	29.0 [23.0;32.0]	11.7 [7.75;15.8]	28.5 [25.5;33.7]	19.5 [9.25;25.0]	34.5 [30.0;40.0]
Carbs	3.48 [1.60;6.15]	0.73 [0.46;1.50]	0.96 [0.46;1.51]	0.92 [0.63;1.15]	0.42 [0.29;1.12]	0.38 [0.19;0.77]
Sugar	1.33 [0.78;2.94]	0.56 [0.49;0.56]	0.56 [0.50;0.81]	1.11 [0.61;1.22]	0.56 [0.56;0.89]	0.56 [0.22;0.83]
Protein	28.0 [14.7;35.2]	32.0 [29.0;37.0]	32.0 [28.9;35.5]	29.0 [28.5;32.0]	28.8 [26.5;32.0]	26.0 [26.0;28.7]
Salt	20.0 [13.5;26.7]	20.0 [16.8;24.8]	27.2 [23.3;31.7]	31.0 [24.6;35.0]	33.3 [28.7;36.7]	30.0 [28.3;33.3]

Median coverage [IQR: p25;p75] of reference intakes from front-of-the pack labelling (reference intakes for adults; 2,000 kcal) of plant-based meats and meats.

Table S3. Coverage of daily intake (%) for nutrients by energy and selected nutrients in adults per 100 ml of PB "pate alternatives", AB pate according to Reference Intakes.

	Plant-based pate	Pate
	N=64	N=16
**Energy**	13.2 [9.80;16.4]	13.4 [12.7;13.9]
**Fats**	29.9 [22.8;44.4]	33.1 [28.9;34.7]
**Saturated fats**	15.2 [10.0;24.0]	43.5 [39.2;45.5]
**Carbs**	2.65 [1.68;3.43]	0.52 [0.49;1.97]
**Sugar**	1.94 [0.56;4.58]	1.28 [1.11;1.44]
**Protein**	9.00 [4.15;13.1]	23.2 [20.8;23.9]
**Salt**	19.8 [16.7;26.7]	30.0 [25.0;31.7]

Median coverage [IQR: p25;p75] of reference intakes from front-of-the pack labelling (reference intakes for adults; 2,000 kcal) of plant-based pate, animal-based pate, plant-based breaded products, plant-based ready dishes, plant-based sweets, and plant-based ice-creams.

Table S4. Coverage of daily intake (%) for nutrients by energy and selected nutrients in adults per 100 ml of PB "dairy product alternatives" and AB dairy products according to Reference Intakes.

	Plant-based yoghurt	Yoghurt	Plant-based cheese	Cheese
	N=12	N=16	N=21	N=16
**Energy**	3.98 [3.67;4.21]	3.75 [2.89;4.80]	15.6 [14.1;18.1]	13.6 [11.0;17.6]
**Fats**	3.00 [2.82;3.07]	3.50 [2.21;4.61]	34.3 [32.1;41.2]	30.1 [23.2;40.8]
**Saturated fats**	1.50 [1.50;2.00]	8.50 [4.25;11.0]	70.0 [26.0;100]	62.5 [56.6;87.1]
**Carbs**	4.33 [3.67;5.18]	2.67 [1.77;4.69]	7.31 [4.23;8.85]	0.48 [0.19;0.78]
**Sugar**	9.28 [6.06;11.5]	7.33 [4.75;13.4]	0.23 [0.00;3.31]	0.72 [0.44;2.25]
**Protein**	7.30 [5.15;7.40]	7.60 [6.60;9.30]	4.00 [1.00;28.4]	34.0 [24.6;50.0]
**Salt**	1.50 [1.25;2.25]	1.83 [1.67;2.00]	26.7 [20.0;36.7]	17.7 [12.9;26.7]

Median coverage [IQR: p25;p75] of reference intakes from front-of-the pack labelling (reference intakes for adults; 2,000 kcal) of plant-based yoghurt, yoghurt, plant-based cheese, and cheese.

Table S5. Nutrient profiling models according to Nutri-Score, NOVA and ECO-Score for AB milks and foods.

	Milk	Meat	Pate	Yoghurt	Cheese
	N=204	N=121	N=16	N=16	N=16
**NUTRI-SCORE**					
**A**	19 (9.31%)	1 (0.83%)	0 (0%)	5 (31.25%)	2 (12.50%)
**B**	184 (90.2%)	6 (4.96%)	0 (0%)	5 (31.25%)	1 (6.25%)
**C**	1 (0.49%)	29 (23.97%)	0 (0%)	6 (37.5%)	1 (6.25%)
**D**	0 (0%)	79 (65.29%)	7 (43.75%)	0 (0%)	12 (75%)
**E**	0 (0%)	6 (4.96%)	9 (56.25%)	0 (0%)	0 (0%)
**Missing**	0 (0%)	0 (0%)	0 (0%)	0 (0%)	0 (0%)
**NOVA**					
**1**	60 (29.41%)	0 (0%)	0 (0%)	0 (0%)	0 (0%)
**2**	0 (0%)	0 (0%)	0 (0%)	0 (0%)	0 (0%)
**3**	17 (8.33%)	1 (0.83%)	1 (6.25%)	3 (18.75%)	11 (68.75%)
**4**	9 (4.41%)	33 (27.27%)	11 (68.75%)	13 (81.25%)	4 (25%)
**Missing**	118 (57.84%)	84 (69.42%)	4 (25.00%)	0 (0%)	1 (6.25%)
**ECO-SCORE**					
**A**	0 (0%)	0 (0%)	0 (0%)	1 (6.25%)	0 (0%)
**B**	23 (11.27%)	0 (0%)	0 (0%)	8 (50%)	0 (0%)
**C**	50 (24.51%)	0 (0%)	1 (6.25%)	4 (25%)	2 (12.50%)
**D**	125 (61.27%)	10 (8.26%)	5 (31.25%)	0 (0%)	13 (81.25%)
**E**	0 (0%)	26 (21.49%)	5 (31.25%)	0 (0%)	1 (6.25%)
**Missing**	6 (2.94%)	85 (70.25%)	5 (31.25%)	3 (18.75%)	0 (0%)

Number of foods with Nutri-Score, NOVA and Eco-Score levels and the percentage of food group per level is indicated between brackets.

Table S6. Nutritional information from the pack labelling, per 100 grams of PB products reported in other studies.

	All meat analogues* N=229	Cheese analogues N=188	Meat analogues N=386	All meat analogues N=168
**Energy**	198 [155;230]	249.0-288.2	146.5-244.4	161.5-233.9
**Fats**	8.7 [5.8;11.9]	20.5-24.78	5.1-16.0	5.7-11.0
**Saturated fats**	1.3 [0.9;1.6]	14.0-18.1	1.0-3.4	0.8-2.6
**Carbs**	12.5 [5.0;18]	4.5-20.9	4.3-16.9	
**Sugar**	1.3 [0.5;2.6]	0.1-1.9	1.0-2.3	
**Protein**	14.0 [9.8;17.0]	0.9-6.8	15.3-21.3	11.1-20.8
**Fiber**				4.6-5.6
**Salt**	1.2 [0.9;1.5]	0.8-2.1	1.2-1.8	0.7-1.4
	Cutroneo et al. 2022	Pointke et al. 2022	Pointke et al. 2022	Alessandrini et al. 2021

*Median values and interquartile range are indicated [p25-p75], and range mean values from different food groups.

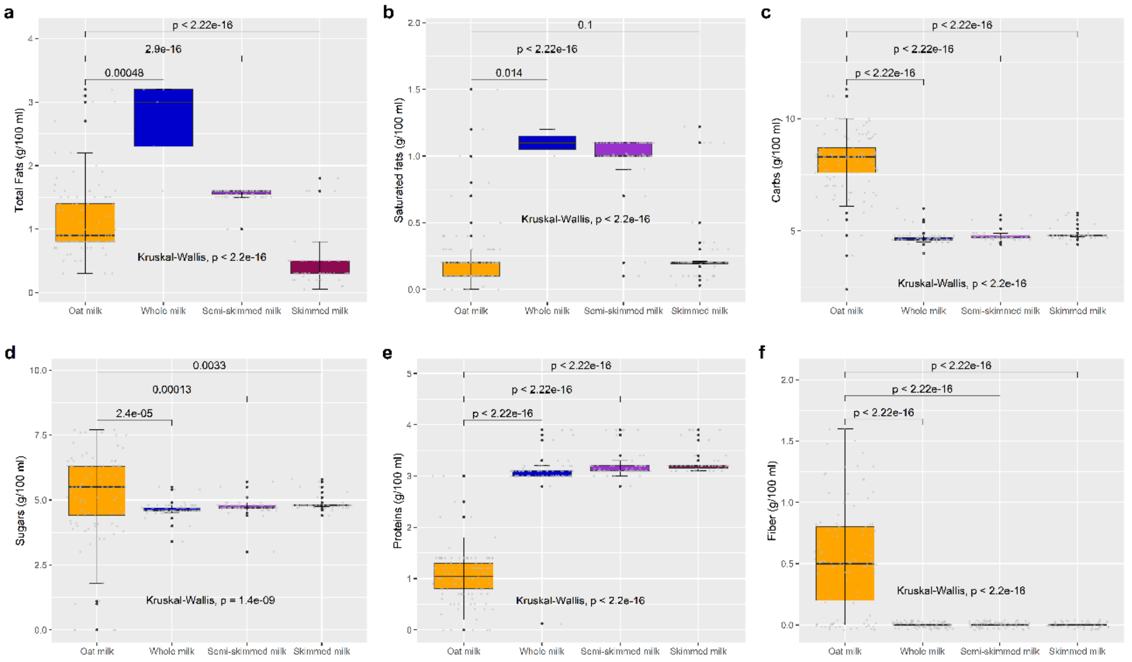

Figure S1. Boxplots of the nutrient composition, per 100 ml, of PB oat "milk" and AB milks. Oat "milk" (yellow colour), as representative of PB "milks", is compared with milks of animal origin (whole milks in blue, semi-skimmed milk in purple, and skimmed milk in red colour). The nutrient composition is shown for total fats (a) and saturated fats content (b), carbs (c) and sugars content (d), proteins (e) and fiber (f) relative to 100 mL milk. P-values are derived from Wilcoxon test (between two groups) and from Kruskal-Wallis tests (between more than two groups).

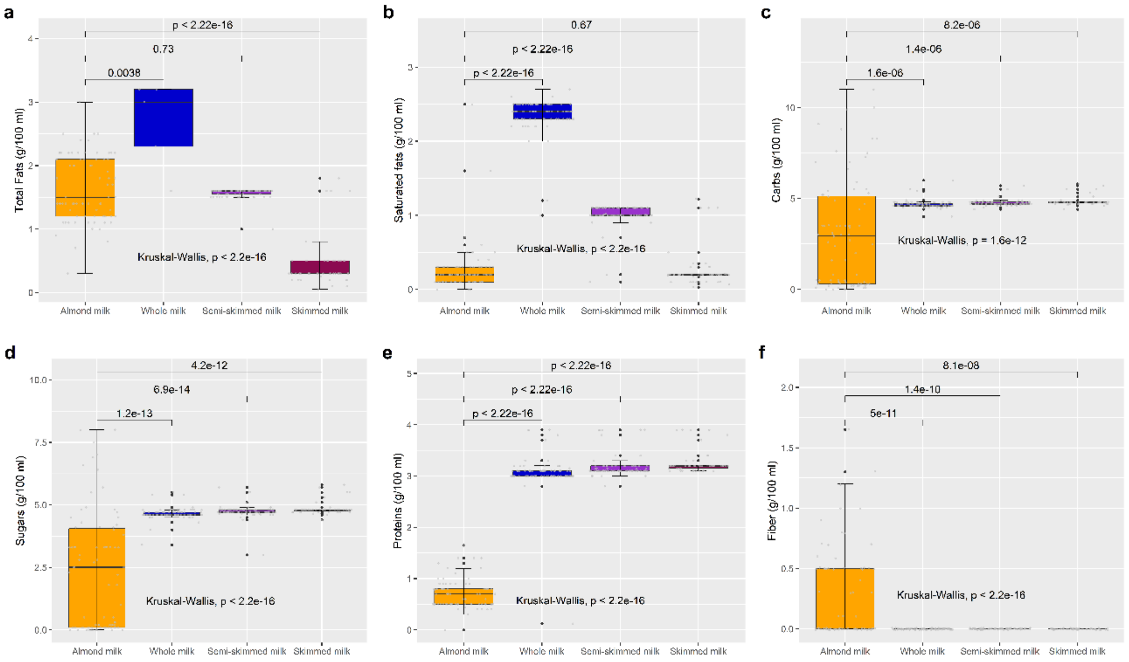

Figure S2. Boxplots of the nutrient composition, per 100 ml, of PB almond "milk" and AB milks. Almond "milk" (yellow colour), as representative of PB "milks", is compared with milks of animal origin (whole milks in blue, semi-skimmed milk in purple, and skimmed milk in red colour). The nutrient composition is shown for total fats (a) and saturated fats content (b), carbs (c) and sugars content (d), proteins (e) and fiber (f) relative to 100 mL milk. P-values are derived from Wilcoxon test (between two groups) and from Kruskal-Wallis tests (between more than two groups).

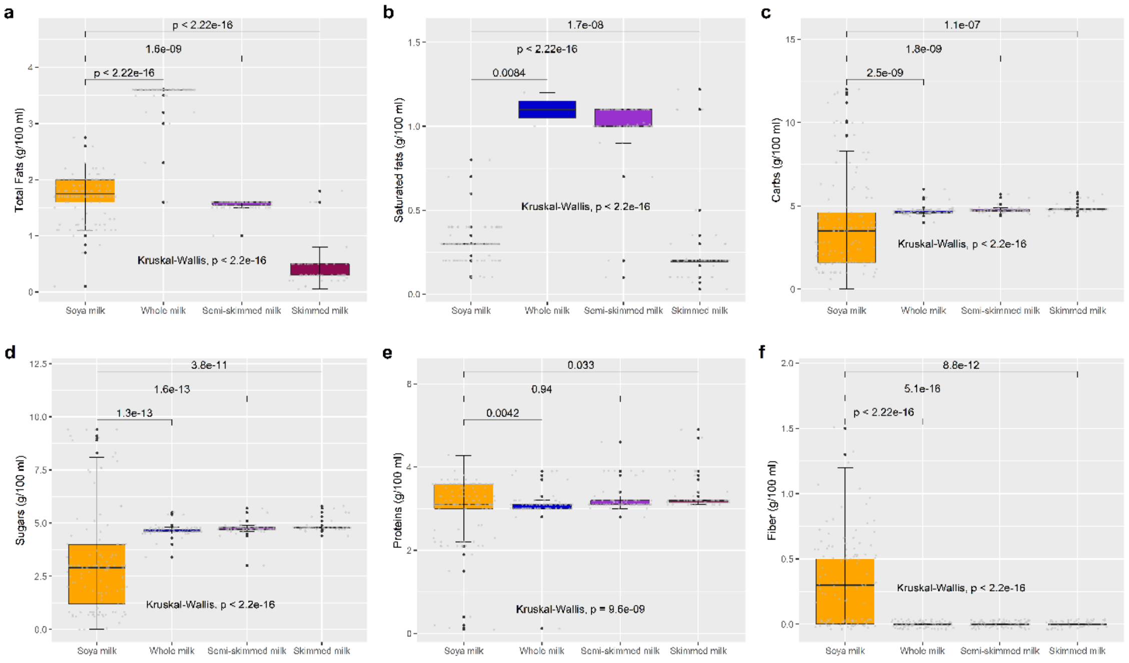

Figure S3. Boxplots of the nutrient composition, per 100 ml, of PB soy "milk" and AB milks. Soy "milk" (yellow colour), as representative of PB "milks", is compared with milks of animal origin (whole milks in blue, semi-skimmed milk in purple, and skimmed milk in red colour). The nutrient composition is shown for total fats (a) and saturated fats content (b), carbs (c) and sugars content (d), proteins (e) and fiber (f) relative to 100 mL milk. P-values are derived from Wilcoxon test (between two groups) and from Kruskal-Wallis tests (between more than two groups).

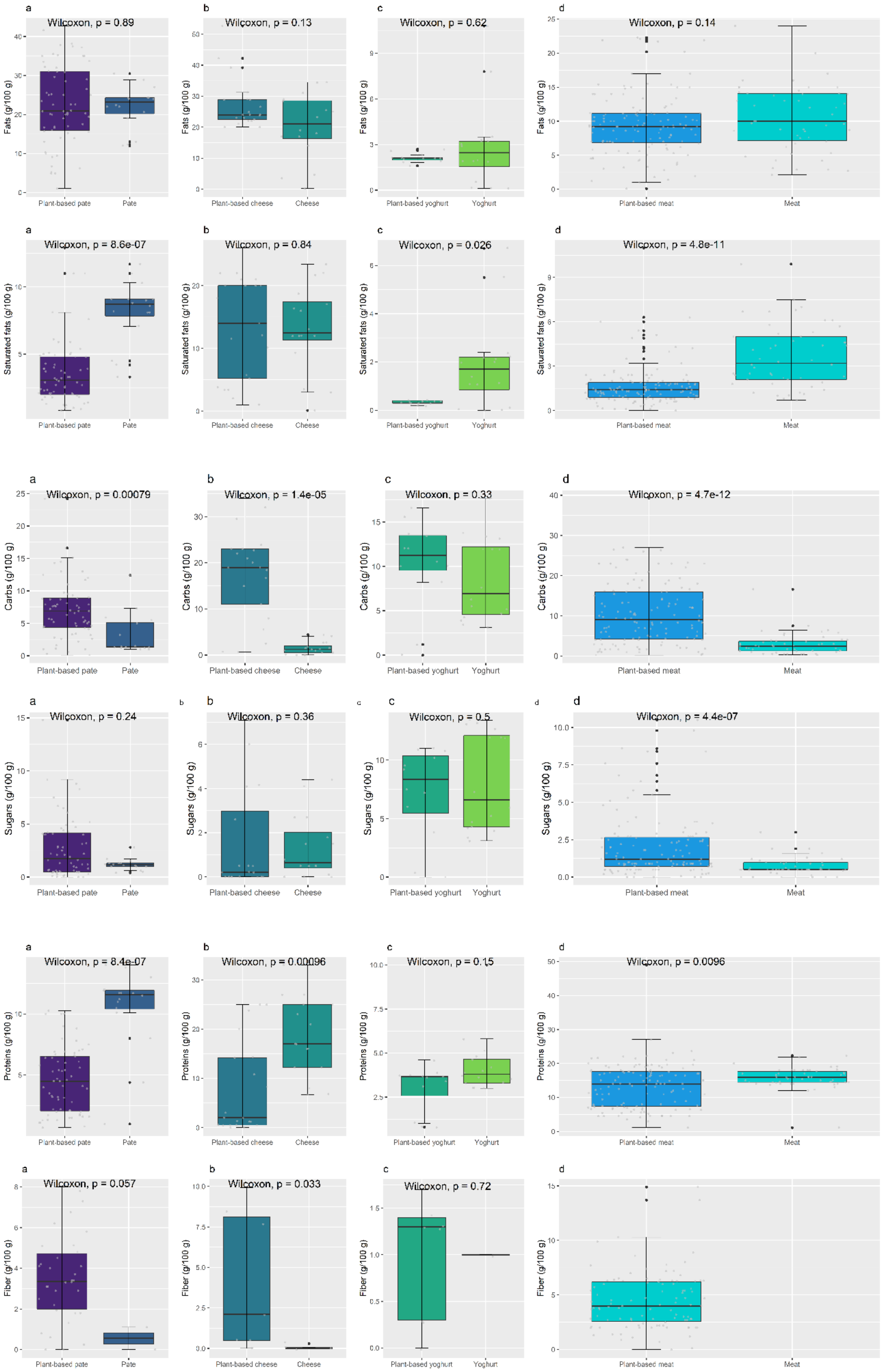

Figure S4. Boxplots of the nutrient composition profiles, per 100 g, of PB and AB foods. PB "pate" (a), "cheese" (b), "yoghurt" (c) and "meats" (d) are compared with some AB foods analogues. The nutritional profile is shown for total fats, saturated fats, carbs, sugars, proteins, and fiber relative to 100 g food. P-values are derived from Wilcoxon tests.
